# Bacteria-inspired nanorobots with flagellar polymorphic transformations and bundling

**DOI:** 10.1038/s41598-017-14457-y

**Published:** 2017-10-26

**Authors:** Jamel Ali, U Kei Cheang, James D. Martindale, Mehdi Jabbarzadeh, Henry C. Fu, Min Jun Kim

**Affiliations:** 1 Department of Mechanical Engineering & Mechanics, Drexel University, Philadelphia, PA 19104 USA; 2 Department of Mechanical and Energy Engineering, South University of Science and Technology, Shenzhen, 518055 China; 3 Department of Mechanical Engineering, University of Utah, Salt Lake City, UT 84112 USA; 4 Department of Mechanical Engineering, Southern Methodist University, Dallas, TX 75275 USA

## Abstract

Wirelessly controlled nanoscale robots have the potential to be used for both *in vitro* and *in vivo* biomedical applications. So far, the vast majority of reported micro- and nanoscale swimmers have taken the approach of mimicking the rotary motion of helical bacterial flagella for propulsion, and are often composed of monolithic inorganic materials or photoactive polymers. However, currently no man-made soft nanohelix has the ability to rapidly reconfigure its geometry in response to multiple forms of environmental stimuli, which has the potential to enhance motility in tortuous heterogeneous biological environments. Here, we report magnetic actuation of self-assembled bacterial flagellar nanorobotic swimmers. Bacterial flagella change their helical form in response to environmental stimuli, leading to a difference in propulsion before and after the change in flagellar form. We experimentally and numerically characterize this response by studying the swimming of three flagellar forms. Also, we demonstrate the ability to steer these devices and induce flagellar bundling in multi-flagellated nanoswimmers.

## Introduction

The field of small scale robotics is developing rapidly, in large part due to the potential for these machines to operate with precision on the cellular and sub-cellular level. The smallest such constructs, synthetic molecular machines^1,2^, can consist of fewer than 20 atoms^3^, however there are a significant number of challenges yet to overcome before they are of any practical use^4^. On the other hand, microorganisms use biomolecular machines to perform myriad useful tasks. For example, in low Reynolds number fluidic environments, many microorganisms propel themselves *via* biomolecular motors which utilize viscous drag. To accomplish this, these organisms often employ some form of nonreciprocal motion^5^. One example of this motion is the oblique beating of flagella and cilia, which protrude from prokaryotes and eukaryotes. This method of swimming has been mimicked in an artificial swimmer composed of streptavidin coated magnetic microparticle chains linked together through biotinylated DNAs, attached to an erythrocyte, and actuated using an oscillating field^6^. Similarly, other swimmers that use flexible metallic nanowires have also been reported^7,8^. However, in nature there is another example of nonreciprocal motion, namely the rotation of helical flagella used by bacteria^9^. In particular, this method of propulsion has been extensively investigated for robotic swimmers, and the design and actuation of biomimetic micro- and nanoscale artificial flagella has been well established^10–13^. Furthermore, potential biological and biomedical applications, including micro transportation^13^, drug delivery^14^, *in vitro* cell manipulation^15,16^, and *in vivo* imaging^17^ have been demonstrated using such devices. While these small scale helical shaped devices have proven to be effective swimmers, many limitations in both their fabrication and actuation ability are still prevalent. In terms of fabrication, most of the reported nano sized helical swimmers have been created using top-down fabrication methods, such as shadow-growth^10^ or direct laser writing^13^, that require specialized equipment and often involve complex fabrication steps. Also, these fabrication methods have been traditionally limited to just inorganic (*i.e*. metals and metalloids) or photoactive polymer materials^18–21^. With respect to actuation, for the nanoswimmers reported thus far, surface coatings have been used to augment additional functionality^14,22^.

However, in comparison to their biological counterpart, artificial bacterial flagella still do not match the structural and mechanical abilities of natural bacterial flagella in many ways. With an inner diameter of ~2 nm and outer diameter of ~20 nm, bacterial flagella are polymeric, self-assembled, hollow nanotubes. Bacterial flagella are much simpler in structure than eukaryotic flagella, and are composed entirely of a single 55 kDa globular protein, flagellin^23^. Flagellin has a number of unusual properties. For example, it can exist in biologically harsh environments, such as extreme pHs (*e.g*. pH 3–11)^24^, and temperatures up to 60 °C^25^, without being denatured. Also, within its molecular structure there exists a beta-hairpin region that undergoes discrete sub-angstrom level conformational changes in response to external stimuli, which in turn causes slight changes in the flagellin structure, leading to large scale changes in the helical pitch and radius of the flagellum^26^. Based on a geometric model, Calladine^27^ predicted that flagella can transform between 12 distinct helical shapes, or polymorphic forms, although not all of these forms have been experimental observed.

The ability to rapidly and reversibly convert between different superhelical forms allows the flagellum to balance external forces that would otherwise cause it to yield or buckle, and lose its rigid form during swimming^28^. Thus far, it has been shown that polymorphic transformations can be induced though environmental changes in pH and ionic strength^29–31^, temperature^24,32^, addition of organic solvents^33,34^, by applying electric fields^35^, or mechanical force^24,36^. The ability of flagella to undergo polymorphic transformation when exposed to a variety of different stimuli gives flagella the potential to be used as both nanoscale sensors and mechanical transducers. These capabilities are not present in previously reported artificial helical nanoswimmers, however there has been a recent report of a sub-millimeter scale soft reconfigurable helical swimmer^37^. Also, in a previous work we developed a microscale swimmer using bacterial flagella, however we were unable steer these devices nor visualize flagella during swimming^38^. Notably, many microorganisms, especially those responsible for infection, utilize the ability to change morphology to effectively infiltrate and bypass physical biological barriers present with host systems. Robotic nanoswimmers that utilize flagella, which have a programmable morphology, could potently mimic this mechanism to navigate complex biological microenvironments. Thus, in an effort to harness the properties of bacterial flagella for both swimming and sensing, we report on the fabrication, visualization, and actuation of shape reconfigurable nanorobotic swimmers.

## Results

### Self-Assembly and Actuation of Flagellar Nanorobots

**Figure 1:**
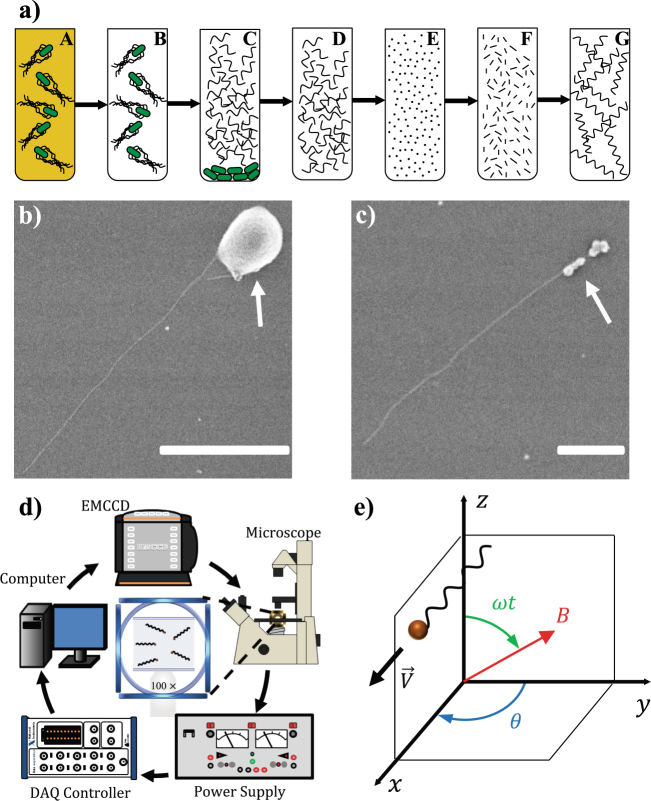
Flagellated nanoparticles and magnetic actuation system. (**a**) Diagram of flagella repolymerization: (A) bacteria are cultured; (B) centrifuged and resuspended in buffer; (C) vortexed to separate flagellar filaments; (D) and isolated through ultracentrifugation; (E) depolymerized by heating; (F) a small portion is repolymerized in to short fragments (seeds); (G) the seeds are added to the original solution of flagellin and repolymerization is carried out for 72 hours. (**b** and **c**) SEM images of flagella attached to magnetic particles (indicated by white arrows); Scale bar is 1 µm in (**b**) and (**c**) 0.5 µm (**d**) Diagram of experimental coil system (**e**) Schematic of flagellar nano swimmer’s motion and the rotating magnetic field.

The design of the nanoswimmers consists of one or more bacterial flagella attached to a superparamagnetic nanoparticle *via* biotin-streptavidin complexes. This design mimics the flagellum-flagella motor complex, however deviates from the bacterial structure in that (1) there is only one flagellum per flagellar motor in bacteria, whereas multiple flagella can be attached to a single nanoparticle, and (2) a universal flexible joint, known as the flagellar hook, is not present in our artificial swimmer. Key to the fabrication of these nanomachines is the ability of flagella to depolymerize into the flagellin homopolymer and repolymerize into long flagellar filaments through a ‘seeded’ polymerization mechanism^39^. By controlling the depolymerization-repolypmerization processes, resultant flagella can be chemically modified such that desired functional groups are present at specific locations along the flagellum. Using this process we produced repolymerized flagella with a short segment of the proximal end functionalized with biotin. To obtain biotinylated flagella, we employed a variation of a method first reported by Asakura *et. al*.^40–42^, and later adapted by others^24,43,44^. For our source of flagella, we used *Salmonella typhimurium* (SJW1103). Flagella were obtained by mechanically shearing flagella from bacteria through vortexing, and were subsequently isolated and washed through centrifugation. The flagella were then depolymerized into flagellin, repolymerized into flagella fragments (~200 nm in length) that were then biotinylated and used as seeds for polymerization of longer flagella (Fig. 1a).

Due to their diameter, direct real time visualization of individual flagella is not possible with traditional bright field microcopy. However, while other methods, including dark-field^45^ and differential interference-contrast microscopy^46^ can be used to resolve flagella, fluorescence imaging has proven to be particularly effective for visualizing moving flagella^47^. Thus, using a previously reported method^48,49^, we fluorescently labeled repolymerized flagella using a succinimidyl ester of Cy3. After labeling, a suspension of flagella was directly added to another solution containing streptavidin coated superparamagnetic nanoparticles (0.005% w/v), and gently agitated for 15 min before visualizing. The biotin functionalized end of flagella self-assembled onto the streptavidin coated surface of the superparamagnetic nanoparticles, resulting in flagellated particles. Magnetic nanoparticles were not visible during experiments, but from scanning electron microcopy (SEM) imaging were approximately 40–400 nm in diameter (Fig. 1b and c). As a result of the polydispersity of the flagella and nanoparticles, we observed flagellated swimmers that consisted of long (>10 µm) and short (<5 µm) flagella, multi-flagellated particles, as well as aggregates of nanoparticles and flagella. Of these various structural forms single flagellated particles were more frequently observed, however coarse control over fabrication of specific geometries may be achieved by adjusting the concentration of mixed flagella and nanoparticles.

Actuation of the flagellar nanoswimmers was accomplished using rotational magnetic fields generated by three pairs of orthogonally positioned electromagnetic coils (Fig. 1d). This coil system was fixed within an epi-fluorescent inverted microscope equipped with a ×100 oil emersion objective and electron multiplying charge coupled device (EMCCD) camera. Magnitude and orientation of the rotational magnetic field was manually controlled *via* a LABVIEW program, as previously reported^50^. While the control system was capable of full 3D motion control, experiments were only conducted in the *xy* plane.

Before conducting experiments with flagellated swimmers, the magnetic responsiveness of superparamagnetic nanoparticles (40–400 nm in diameter), with no flagella, was analyzed. Nanoparticles, dispersed in a solution of 40% ethylene glycol, were rotated in-plane at a series of increasing external field frequencies. For these experiments the magnitude of the magnetic field was held constant at 15 mT, and brightfield images of particle rotation were acquired at a rate of 500 Hz. The average rotation rate of geometrically anisotropic particles was obtained by applying a Fourier transform on their intensity fluctuations, as previously reported^51^. As shown in Fig. S1, the rotational response of a nanoparticle linearly increases with the applied field up to approximately 30 Hz, after which a non-linear decrease in rotation was observed. The transition from synchronous to asynchronous rotation has been established to be a result of the phase-lag between the dipole moment of the particle and applied field increasing to more than 90°, above which point the magnetic torque is not sufficient enough to completely overcome the viscous drag^51^. Therefore, to ensure that flagellated nanoparticle rotation occurred in the synchronous regime, a limit on the rotational frequency was set to below 30 Hz.

### Swimming Characterization

To determine the swimming effectiveness of flagellated nanoparticles with different helical geometries, experiments were performed for swimmers in normal, curly, and coiled forms. From florescence image measurements, it was observed that normal and curly form swimmers had similar lengths, while swimmers in the coiled form were overall shorter (Table 1). Experiments were conducted by placing solutions containing swimmers in a cylindrical polydimethylsiloxane (PDMS) chamber, 2 mm in diameter and 2 mm in height, sealed on both sides by no.1 thickness glass coverslips, and placed in the center of the coil system. For comparison, only swimmers with one observed flagellum were tracked and analyzed. Also, it is important to note that the normal and coiled forms are left handed helices, while the curly form is right handed, which causes the curly form to move in the direction opposite of the normal and coiled forms when exposed to an identically oriented magnetic field. Normal forms could be transformed into coiled and curly forms by immersing in aqueous solutions of 40% (v/v) ethylene glycol^33^ or 50% (v/v) dimethyl sulfoxide (DMSO)^52^, respectively. For the normal form, 45% (v/v) DMSO was used. Although the dynamic viscosity of these fluids is not identical, they are within 0.3 cP of each other^53,54^. These solutions were used to create Newtonian fluids (*i.e*. no fluid elasticity) with dynamic viscosities similar to human blood at physiological temperatures^55^, but are not biocompatible and therefore not suitable environmental stimuli for biological applications. However, other biocompatible environmental stimuli, such as ionic concentration/pH, temperature, and mechanical stimuli can be used to the same effect^24^. All of the flagella in our swimmers are capable of such transformations as demonstrated in Movie S1, where floating coiled flagella are transformed to extended forms due to an increase in organic solvent concentration. The rate of transformations observed in the movie occur on the order of seconds, however when rapidly immersed in aqueous solutions containing organic solvents, the conformational change of flagella between different forms occurs much more rapidly, similar to the rapid (~10 ms) transformations observed in bacteria^56^. Therefore the main effect of such an environmental stimulus is due to differences in propulsion for the form before and after the stimulus. Hence to explore the response to rapidly applied external stimuli, we characterize the swimming behavior for three different helical forms. Also, it’s important to note that while flagella are very thin, they are extremely stiff, with an elastic modulus estimated to be in excess of 10^10^ N/m^2^ ^57^, and thus Brownian fluctuation in their conformations are not normally observed. Furthermore, since the relative importance of translation and rotational Brownian motion is determined by the largest length scale (*i.e*. length of the swimmer) rather than the thickness, flagella act as colloidal particles.

**Table 1:** Measured lengths and turns of single flagellar nanoswimmers.

Swimmer Number	End-to-End Length (µm)			Total Contour Length (µm)			Total Number of Turns		
	Normal	Curly	Coiled	Normal	Curly	Coiled	Normal	Curly	Coiled
1	6.65	5.82	2.82	7.27	6.48	5.28	2.81	4.86	1.49
2	6.72	6.22	2.63	7.36	6.95	5.15	2.84	5.21	1.45
3	8.35	6.05	2.12	9.05	6.65	4.34	3.49	4.99	1.22
4	5.20	4.78	2.25	5.89	5.27	4.54	2.27	3.95	1.28
5	4.65	5.06	2.30	5.27	5.75	4.62	2.03	4.31	1.30

**Figure 2:**
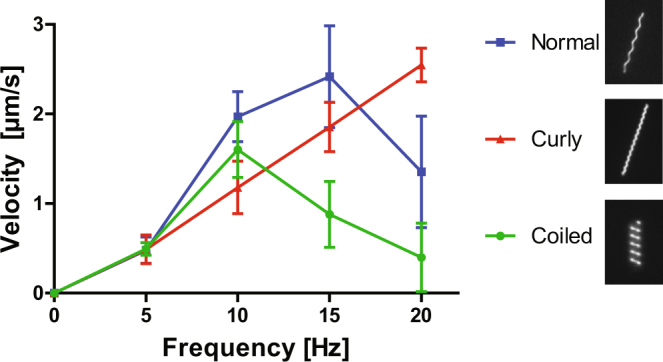
Swimming characterization. Velocity profiles of flagellar nanoswimmers plotted against rotation frequency.

The velocity of nanoswimmers, with different helical forms, was plotted against rotational frequency (Fig. 2). The external rotational field was increased from 0 to 20 Hz, with an increment of 5 Hz. Variations in the velocity-frequency profiles are primarily due to (1) the geometric differences of the different helical forms (see Table 2) and (2) variations in flagella length (Table 1) and size of the magnetic particle attached to the flagellum. All swimmers monotonically increase in velocity with increasing frequency, until a point (*i.e*. step-out frequency)^13^ at which they begin to rotate asynchronously with the external field, after which rapid decrease in swimming speed is observed with greater applied field frequency. This behavior is the result of the geometry of the different flagella increasing the viscous drag, to varying degrees, of the magnetic nanoparticle to which they are attached. From 0 to 5 Hz all three swimmers move at approximately the same speed. However, from 5 to 10 Hz the swimmers’ velocity profiles begin to deviate from each other. For the curly form the velocity profile retains a linear increase in slope, a trend that continues up to 20 Hz. The coiled swimmers have a nonlinear increase in velocity from 0 to 10 Hz, while normal swimmers have a nonlinear profile from 0 to 15 Hz. It is apparent from Fig. 2 that the coiled form has a step-out frequency around 10 Hz, and the normal form at approximately 15 Hz. While curly swimmers are slightly slower than the other forms at 10 Hz, its step-out frequency is located above 20 Hz, and thus has the potential to reach higher swimming speeds. Also, noting that previous theoretical calculations estimated that the optimal pitch angle is approximately 45° ^58^, step-out first occurs for coiled swimmers which have a pitch angle farthest from 45°, followed by normal, and finally curly, which is closest to the optimal angle.

**Table 2:** Helical parameters of flagellar polymorphs.

Polymorphic Form	Handedness	Helical Pitch (µm)	Helical Radius (µm)	Pitch Angle (deg.)
*Normal*	Left	2.23	0.21	31
*Coiled*	Left	0.81	0.55	63
*Curly*	Right	1.14	0.11	40

Aside from the propulsion observed for the normal, curly, and coiled flagellar forms, for single flagellated swimmers we did not observe other types of dynamic behavior (*i.e*. tumbling and precession) observed in abiotic nanopropellesrs^59^. One possible reason that these dynamics were not observed in our swimmers may be because at lowest frequencies investigated (5 Hz) swimmers were already in the propulsion regime of motion. Also, considering that our helices are thinner than previously reported artificial helices and have a larger aspect ratio, their tumbling should be confined to smaller frequencies^60^. In addition, it should also be noted that the nanopropellers are usually coated with a ferromagnetic material along their entire length, while the magnetic component is localized to one end of our swimmers (superparamagnetic nanoparticle) and the actual helix (flagellum) is not magnetized. These differences may have also contributed to observed dynamic behavior.

### Numerical Analysis

**Figure 3:**
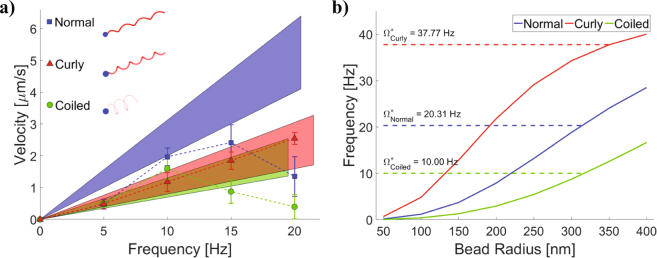
Numerical analysis. (**a**) Colored wedges show the range of velocity-frequency response for swimmers with each polymorphic form across the range of bead sizes, calculated using the method of regularized Stokeslets. Dashed lines and symbols are experimental data from Fig. 3. (**b**) For each polymorphic form, the step-out frequency calculated as a function of bead size assuming constant magnetization per volume. The dashed lines show the average step-out frequency for each curve. The magnetization per volume is chosen to set Ω*Coiled to be 10 Hz, and our calculation predicts the ordering of step-out frequencies observed experimentally.

We performed a numerical study of such swimmers to clarify the effects of flagellar geometry on the swimmers. The method of regularized Stokeslets is used to model the swimmer as a rigid body^61,62^. The swimmer consists of a spherical head with radius varying from 40–400 nm to represent the range of possible magnetic bead sizes observed in the experiments, along with a single rigid flagellum with an appropriate tapering region such that the flagellum and head meet at a single contact point^63^. The regularized Stokeslet spacing and blob size are set using optimal parameters which depend on the flagellum geometry^64^ for each polymorphic configuration taken from Table 1. The normal, coiled, and curly flagella are modeled with 3.5, 2.5, and 4.5 turns, respectively, as measured by image analysis of the swimmers. Pictorial representations of the swimmers with a bead radius of 200 nm are shown in the inset in Fig. 3a.

In our numerical model, we assume force-free conditions on the swimmer and prescribe a constant torque in the *x*-direction (the direction of the helical axis). For a rigid swimmer with a given geometry, these inputs are linearly related to the translation velocity $V$ and the rotation vector Ω *via* a 6 × 6 mobility matrix. The average swimming speed is *V* · $\widehat{{\rm{\Omega }}}$ ^63^. From this, we calculate the ratio of swimming speed to rotation rate, *i.e*., the slope of the velocity-frequency response in the experimental results from Fig. 2. This calculation is a good approximation of the actual swimming speed so long as the precession angle of the swimmer remains small^60^; we have checked that the precession angle is less than 2.75° for all calculated cases. In Fig. 3a we plot as colored wedges the range of slopes which are observed in the numerical study as the bead radius ranges from 40–400 nm. The numerically calculated slopes are comparable to the experimental data from Fig. 2 (replotted in Fig. 3a). The experimental slopes tend to be slightly less than the calculated slopes, which may be attributed to some swimmers in the sample going beyond step-out frequency and to errors in the measured length of flagella used in the calculations. For example, to reduce the slope of the curly swimmer by 25% requires shortening it by a third.

The step-out frequency is determined by when the external torque required to rotate the swimmer at that frequency is equal to the maximum torque capable of being produced by a given moment and field strength. Since we do not know the moment strength, we cannot calculate the absolute step-out frequencies, but we can calculate relative step-out frequencies by assuming that every bead has the same (unknown) magnetization per volume. Calculating the rotation frequency for torques proportional to volume produces a range of step-out frequencies for each polymorphic configuration of the flagellum (Fig. 3b), in which most of the variation is due to the range of bead-sizes. Averaging over the range of bead sizes yields a mean step-out frequency (dashed lines in Fig. 3b), where the magnetization per volume is chosen such that the average step-out frequency for the coiled form is 10 Hz, matching that of Fig. 2. With this choice, we predict the relative ordering of the mean step-out frequencies. From the numerical results, we see that the step-out frequency of the coiled form is the smallest, followed by the normal form, with the curly form having the highest step-out frequency. This is consistent with the experimental results of Fig. 2. Note that we take the mean of step-out frequencies, as opposed to Fig. 2 which gives a single step-out frequency found from the mean velocity-frequency response of several trials. However, from the experimental data it is difficult to calculate a mean of step-out frequencies since, as expected from our model, due to bead size variation each set has at least one swimmer with a step-out frequency which is greater than the measured range. Nonetheless, the *measurable* experimental step-out frequencies follow the same ordering trend as the step-out frequencies obtained from the mean velocity-frequency response. The point at which the maximum torque possible from the field dipole interaction (*m* × *H*) is equal to the rotational drag torque, (*i.e*. step-out frequency) is inversely proportional to viscosity, and therefore in general higher viscosities would yield lower step out frequencies. However, since the different forms analyzed here are in solutions with viscosities within 15% of each other, that viscosity variation will not change the ordering of the step-out frequencies. To summarize, step-out frequencies are strongly affected by the form of the flagella; the coiled form has the largest helical radius, hence largest rotational resistance and lowest step-out frequency.

### Hookless flagellar bundling

**Figure 4:**
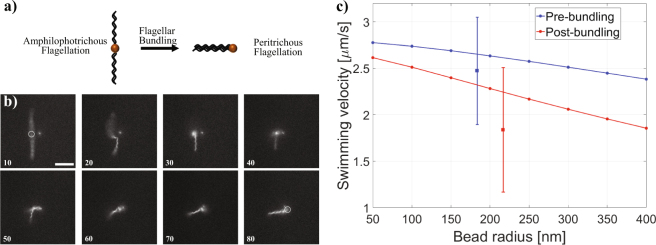
Demonstration of flagellar bundling. (**a**) Diagram of flagellar bundling. (**b**) Successive fields shown at 24 Hz (deinterlaced; total time span, 2.92 s); numbers refer to fields, *i.e*., images obtained at 24 Hz. White outline in fields 10 and 80 represents approximate location of magnetic nanoparticle. Scale bar represents 10 µm. (**c**) Velocities of the flagellar nanoswimmer before and after bundling. Square symbols represent experimental values for averaged swimming speeds. Circles represent values calculated using the method of regularized Stokeslets.

Due to the nature of the self-assembly processes involved in both the flagellar repolymerization and particle attachment, we observed a heterogeneous population of swimmers, most of which consisted of one to three flagella of lengths ranging from 5–20 µm. From our observations we could distinguish swimmers with distinct flagellar arrangements. Swimmers with only one flagellum are reminiscent of monotrichous bacteria, while swimmers with multiple flagella are similar to peritrichously flagellated bacteria such as *Escherichia coli* and *Serratia marcescens*. However, note that the bodies of the artificial swimmers are an order of magnitude smaller than bacteria. Also, occasionally we also observed swimmers that appeared to be amphilophotrichous (*i.e*. tufts of flagella on opposing sides of the nanoparticle, Fig. S1). One such swimmer, rotating in a fixed external field frequency of 8 Hz, is shown in Fig. 4 and Movie S2. The swimmer consists of a total of four flagella, initially with two flagella located on opposite sides of the nanoparticle. Two of the flagella are approximately 11 µm in length, while the other two are 10 and 15 µm long. Initially the swimmer moves in the upward direction, and then the orientation of the field changes from counterclockwise to clockwise (*i.e*. the direction of rotation is changed 180°) and the swimmer moves downward. The average swimming velocity during this period is 2.47 µm/s. A few seconds after this, the direction of rotation is changed 90°, and the swimmer moves in the direction perpendicular to its original swimming direction which causes its flagella to bundle. Post-bundling, the average swimming velocity is 1.84 µm/s and the end-to-end length of the bundled flagellum is approximately 10 µm. No flagella appear to detach from the particle after bundling, however some of the bundled flagella may be wrapped around the nanoparticle causing the flagella bundle to appear shorter than some of the individual flagella seen pre-bundling. While we know of no bacteria which have been observed to switch between flagellation states, the processes of flagellar bundling, albeit with no observable polymorphic transformation, appears to resemble the bundling process that occurs between the run and tumble cycles of peritrichously flagellated bacteria. However it should be noted that in bacteria, bundling occurs when all flagellar motors are rotating counterclockwise, resulting in a run; when one or more flagella rotates clockwise the bundle unbundles, and a tumble occurs. Unlike bacteria, we did not observe unbundled flagella to bundle or *vice versa* when changing the rotation direction counterclockwise or clockwise, instead when changing the direction of rotation 180° the swimmer simply reversed swimming direction. There are three main differences between our swimmer and bacteria that are underlying the differences observed in bundling-unbundling behavior: (1) in bacteria each flagella is attached to its own flagellar motor that rotate independently of each other, while in nanoswimmers all flagella are attached to the same ‘motor’(magnetic nanoparticle), (2) bacteria rotate their flagella an order of magnitude faster that that used in our experiments, (3) bacteria have a flexible hook connecting the flagellum to the flagellar motor, which is absent in our experiments. Also note that the presence of a flexible hook is necessary for bundle formation in peritrichous flagellates^65^, and thus the observed hookless bundling, possibly through flagellar buckling instability^66^, may be an alternative bundling mechanism for multi-flagellated artificial swimmers.

In order to model this experiment, we use the method of regularized Stokeslets to discretize the swimmer pre- and post-bundling as in the previous numerical study. Pre-bundling, we assume that the two helical filaments on each side of the magnetic bead are tightly bundled with end-to-end length 11 µm and share the same helical axis. Bundled flagella are modeled as a single flagellum with an effective filament radius which depends on the number of filaments in the bundle and the compactness of the bundle^67^. Assuming tight packing for two filaments of radius $a$ gives an effective filament radius of $\tilde{a}=2a$. We note that measurements of swimming velocity vary by <5% if this effective radius is doubled to $4a$, meaning our results are not sensitive to this parameter. Post-bundling, all four filaments are bundled tightly with end-to-end length 10 µm and we use an effective radius of $\tilde{a}=2.4a$. The rotation rate is set at 8 Hz to match experimental parameters, and the bead radius is varied from 50–400 nm. We plot the results of swimming velocity pre- and post-bundling as a function of bead radius in Fig. 4c. The experimentally measured mean swimming velocities are presented with error bars representing the standard deviation of the measurements. The mean swimming velocity measured in the experiments decreases approximately 34% post-bundling. In our numerical study, we observe a decrease in swimming velocity for all bead sizes in our range, with larger differences for larger bead sizes. For an example of a typical bead size of 300 nm, we find that the swimming velocity decreases by 22% post-bundling.

The hookless bundling seen in our experiments raises the question of whether it requires compliance in the flagella-bead attachment or may be achieved solely by bending the flagellum. Our numerical model allows us to estimate the bending forces on the filaments as the swimmer transitions from pre- to post-bundling configuration. To do this, we change the rotation direction of the pre-bundling swimmer by 90° and calculate the cumulative force and torque on the filament at the contact point with the bead. In order for bundling to occur, the filaments must bend 90° over a bead radius taken to be 300 nm. To estimate the bending stiffness, we use the Kirchhoff rod theory^68^ to fit a bending stiffness that allows an initially straight filament to bend 90° over a 300 nm distance for the calculated applied force and torque (6.77 pN, 52.78 pN µm, respectively) at the contact point and obtain an estimate of 15.5 pN µm^2^. For comparison, Darnton and Berg^24^ performed force-extension measurements on bacterial flagella of different polymorphic forms and estimated the bending stiffness to be 3.5 pN µm^2^. Our estimate shows that even with a larger bending stiffness than previously measured, bundling could be due to hydrodynamically forced bending of the flagellum itself, although rotation of the attachment point cannot be ruled out by this estimate.

### Steering

**Figure 5:**
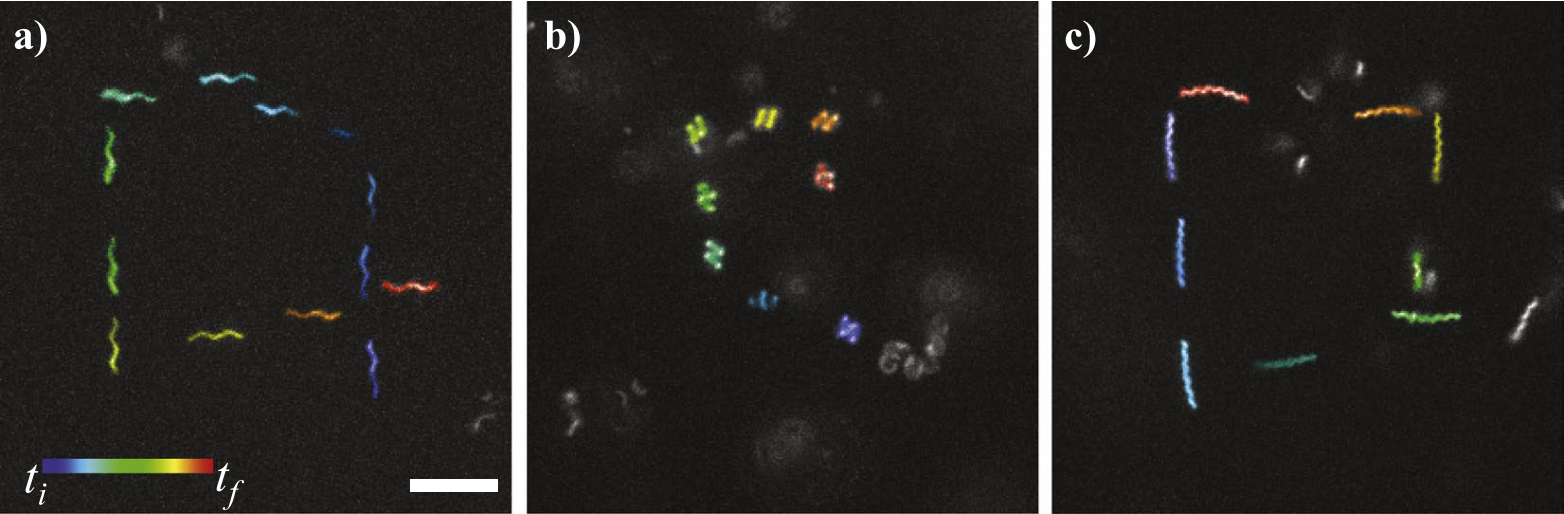
Steering of flagellar nanorobots. Trajectory of (**a**) normal, (**b**) coiled, and (**c**) curly swimmers. Scale bar is 5 µm.

In addition to determining the velocity profiles of the flagellar nanoswimmers, experiments designed to demonstrate the steerability of the swimmers were conducted. For steering, the frequency of the external magnetic field was held constant, while turning of the robot was accomplished by manually adjusting the direction of rotation ($\theta$) in eqs (1) and (2). Figure 5 shows the tracking of a normal, curly, and coiled swimmers. Each swimmer was manually controlled to move in a square path (Movie S3). Note that the swimmers occasionally move in and out of the focal plane, due to a significant amount of Brownian motion. For the normal and curly swimmers, the frequency was held at 3 Hz, and resulted in average swimming speed of 0.3 µm/s. For the coiled swimmer, the frequency was set at 8 Hz, and resulted in average swimming speeds of approximately 1 µm/s, which is in agreement with the velocity profile in Fig. 2.

## Discussion and Conclusions

Here, in an advancement of our previous work^38^, we have demonstrated the ability to fabricate, visualize, and actuate fuel-free stimuli-responsive nanoswimmers consisting of repolymerized bacterial flagella and magnetic nanoparticles. A few aspects of these robots are potently advantageous compared to previously reported swimmers. First, because of the simple fabrication method, the ‘body’ of the swimmer can be easily changed by using magnetic nanoparticles of different size or composition. Also, because the fabrication process relies on self-assembly, large scale batch production of these nanorobots is possible. In terms of swimming, the ability to change polymorphic form and have a relatively soft structure^69^, could make these swimmers particularly adept at navigating^70^ heterogeneous confined viscoelastic fluids which are ubiquitous in nature. By combining the ability to rapidly change morphology with autonomous feedback control, advanced navigation strategies can be developed such that swimmers can actively adapt to surrounding dynamic biological environments on demand in real-time. Finally, by visualizing polymeric transformations, in response to changes in the fluidic environment, these swimmers can serve as nanoscale probes.

## Methods

### Flagella Repolymerization

Bacterial flagella were isolated, depolymerized, and repolymerized as previously reported^24,38,44^. First, a log-phase culture of *Salmonella typhimurium* (SJW1103) was obtained by an overnight culture of the bacteria in a modified LB broth (1% tryptone(w/v), 1% yeast extract(w/v), 0.1% glucose(w/v), in 10 mM potassium phosphate buffer). Flagella were isolated from bacterial bodies by vigorous vortexing for 10 min, isolated by centrifugation at 10,000 × *g*, resuspended in phosphate buffer, and pelleted by centrifugation at 100,000 × *g*. Next, isolated flagella were depolymerized into flagellin monomers by heat treatment for 15 min at 65 °C. A portion, ~10% (v/v), of the supersaturated flagellin solution was then added to an equal volume of 2 M Mg_2_SO_4_ and allowed to polymerize for 2 hours at room temperature to generated flagellar fragments (‘seeds’). The short (>200 nm) flagella fragments were washed of salt though centrifugation, and then functionalized with biotin *via* a pegylated N-hydroxysuccinimide ester as per the manufacturer’s instructions. Finally, to obtain flagellar filaments with biotin at one end, the biotinylated seeds were added back to the original supersaturated flagellin solution and allowed to polymerize for three days.

### Fluorescent Labeling

Repolymerized flagella were pelleted by centrifugation at 80,000 × g and resuspended in 10 mM potassium phosphate buffer (pH 7.5). To this solution an equal volume of reconstituted Cy3 dye, in an aqueous solution of 0.1 M sodium bicarbonate, was added. The solution was allowed to incubate at room temperature for 3 hours. Excess dye was removed through centrifugation and the flagella pellet was gently resuspended in phosphate buffer. Labeled flagella were subsequently stored at 4 °C until use.

### Scanning Electron Microscopy

A drop of solution, containing flagella and superparamagnetic nanoparticles dispersed in deionized water, was dispensed onto a clean silicon wafer chip and incubated for five minutes. The drop was gently removed and the silicon chip allowed to completely dry. A thin film (<1 nm) of Pt/Pd was sputtered on to samples before imaging. Images were acquired *via* a Zeiss Supra 50VP field emission scanning electron microscope operated at an acceleration voltage of 1 kV.

### Magnetic Control System

Actuation of the flagellar nanoswimmers was accomplished using rotational magnetic fields generated by three pairs of orthogonally positioned electromagnetic coils (Fig. 1d). This approximate Helmholtz coil system was affixed to a motorized stage that was embedded within a Nikon Ti-E epi-fluorescent inverted microscope equipped with a $\times$100 oil emersion objective and Andor iXon 987 EMCCD camera. Images were acquired at a rate of 24 Hz. The magnitude and orientation of the rotational magnetic field were manually controlled *via* a LABVIEW program, as previously reported^50^. While the control system was capable of full 3D motion control, experiments were only conducted in the *xy* plane. This was accomplished by inputting sinusoidal AC inputs, 90° out of phase, into coil pairs, such that the generated magnetic field (${\boldsymbol{B}}$) could be described as

1 $${\boldsymbol{B}}=[\begin{array}{c}Bsin(\theta )cos(\omega t)\\ Bcos(\theta )cos(\omega t)\\ Bsin(\omega t)\end{array}].$$

where $B$, $\omega$, $\theta$, and $t$ represent the amplitude of the rotating magnetic field, rotational frequency of the field, direction of rotation, and time, respectively. The rotational field synchronously operates orthogonally to the static field. The rotation axis is a unit vector perpendicular to the plane of the rotational field, and can be described as

2 $$\hat{{\boldsymbol{n}}}=[\begin{array}{c}cos(\theta )\\ sin(\theta )\\ 0\end{array}].$$

A representation of eqs (1) and (2) is shown in Fig. 1e.

## Electronic supplementary material

Supplementary Movie 1

Supplementary Movie 2

Supplementary Movie 3

Supplementary Information
