# Supplementary material for: Bacteria-inspired nanorobots with flagellar polymorphic transformations and bundling
Source: Sci Rep. 2017 Oct 26;7:14098. doi: 10.1038/s41598-017-14457-y (PMC5658443; doi:10.1038/s41598-017-14457-y)
Supplement: Supplementary file 4 — Supplementary Information [file 41598_2017_14457_MOESM4_ESM.docx]

**Supplementary Information**

Bacteria-inspired nanorobots with flagellar polymorphic transformations and bundling

Jamel Ali^1^, U Kei Cheang^2^, James D. Martindale^3^, Mehdi Jabbarzadeh^3^, Henry C. Fu^3^, and Min Jun Kim^4,*^

**Supplementary Movie 1.** Shown are two flagella undergoing polymorphic transformations, from a coiled form to an extended form. Flagella are immersed in a solution containing water and a low vapor pressure organic solvent. As water evaporates, the effective concentration of organic solution increases. This induces the polymorphic transformations. The rate of transformation varies due to differences in water evaporation rates. The scale bar is 5 µm.

**Supplementary Movie 2.** A magnetic nanoparticle with tufts of flagella on opposing sides is observed to swim. When the orientation of the applied externally generated magnetic field is changed from counterclockwise to clockwise, the swimmer swims in the opposite direction. The direction of rotation is then changed by 90°, and the swimmer moves in the direction orthogonal to its original swimming direction. This causes its flagella to come together and form a bundle. The scale bar is 5 µm.

**Supplementary Movie 3.** Videos of three monotrichously flagellated swimmers being steered in a square pattern are shown. Two of the flagellar forms, curly and coiled, were induced by the addition of an organic solvent. The scale bar is 5 µm.


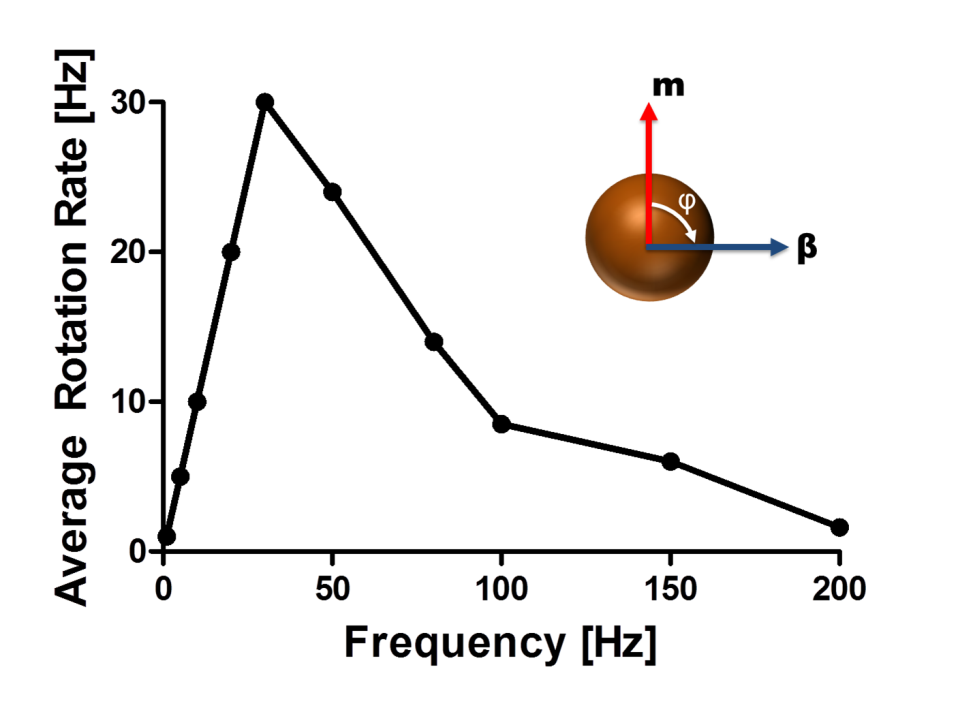


**Fig. S1. Frequency response of a superparamagnetic nanoparticle.** Rotation occurs in a uniform 15 mT field. Asynchronous rotation, i.e. phase-slipping, occurs above 30 Hz. Insert: a schematic showing the phase lag angle φ, formed between the particle dipole moment m and the rotating external field β.


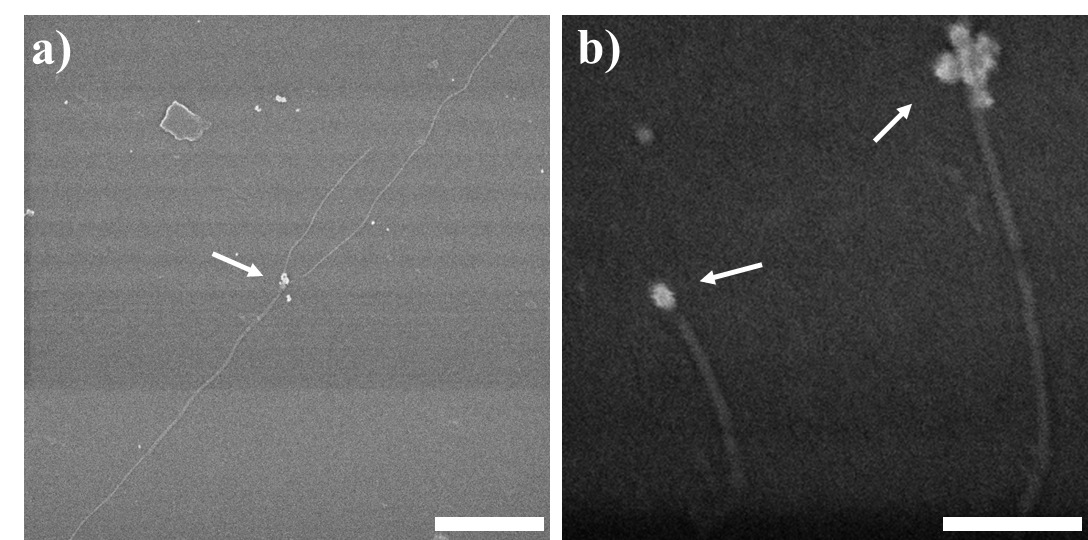


**Fig. S2. SEM images of flagella attached to magnetic nanoparticles.** Nanoparticles indicated by white arrows; Scale bar is 1 µm in (a) and (b) 0.2 µm.


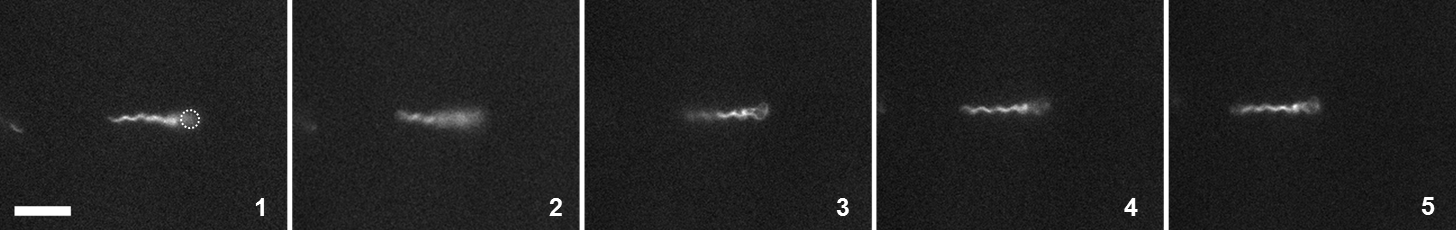


**Fig. S3. Successive fields of swimmer propelled by a flagellar bundle consisting of four flagella.** Images taken at one second intervals. White outline in field 1 represents approximate location of magnetic nanoparticle. Scale bar represents 10 μm.


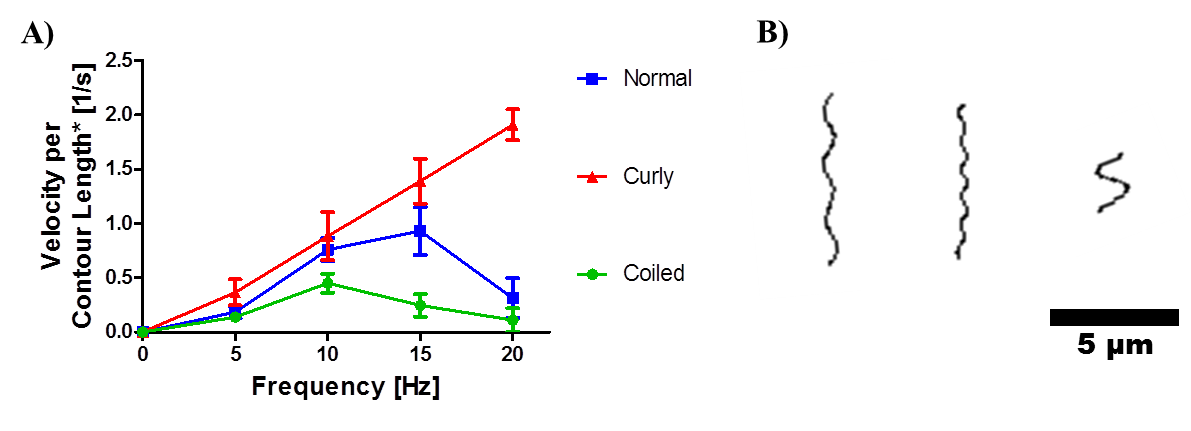


**Fig. S4. Normalized velocity and traces of flagellar nanoswimmers.** (a) Velocity of flagellar nanoswimmers normalized against contour length plotted against rotation frequency. *The contour length ($L$) used here is the length contained in one helical pitch defined by $L^{2}=P^{2}+\left( \pi D \right)^{2}$ , where $P$ is the helical pitch and $D$ is the helical diameter. (b) Smoothed traces of flagellar nanoswimmers. Flagella shown from left to right are in the normal, curly, and coiled polymorph.

**Table S1. Swimming speed comparison of nanohelical swimmers at various rotational frequencies.** Swimming speed values were obtained by extrapolating data from the linear portion of velocity-frequency plots. Except for the flagellar nanoswimmer, swimmer data was obtained in water or buffer solutions ($\mu\sim1 \mathrm{cP}$).

| **Swimmer** | Length | Helix Thickness | Speed at 10Hz | Speed at 20Hz | Speed at 50Hz | Speed at 100Hz |
| --- | --- | --- | --- | --- | --- | --- |
|  | (µm) | (µm) | (µm/s) | (µm/s) | (µm/s) | (µm/s) |
| Flagellar Nanoswimmers (Curly) | ~5.5 | 0.02 | 1.2 | 2.5 | 6.4 | 12.8 |
| Nanovoyager^S1^ | 5.4 | 0.5 | 1.2 | 2.4 | 6.0 | 12.1 |
| Helical Nanostructures^S2^ | 4.5 | 0.4 | 0.6 | 1.3 | 3.6 | 7.4 |
| Nanostructured Propellers^S3^ | 1.5 | 0.2 | 6.9 | 9.1 | 15.5 | 26.3 |
| *E. coli*^S4^ | 10 | 0.02 | - | - | - | 10.0 |

**References**

S1. Venugopalan, PL., et al. Conformal cytocompatible ferrite coatings facilitate the realization of a nanovoyager in human blood. *Nano Lett.* **14**, 1968-1975, (2014).

S2. Ghosh, A. & Fischer, P. Controlled propulsion of artificial magnetic nanostructured propellers. *Nano Lett.* **9**, 2243-2245, (2009).

S3. Ghosh, A., Paria, D., Singh, H. J., Venugopalan, P. L. & Ghosh, A. Dynamical configurations and bistability of helical nanostructures under external torque. *Phys. Rev. E: Stat., Nonlinear, Soft Matter Phys.*  **86**, 031401, (2012).

S4. Patteson, AE., Gopinath, A., Goulian, M. & Arratia, PE. Running and tumbling with *E. coli* in polymeric solutions. *Sci Rep.* **5**, 15761, (2015).
